# Supplementary material for: The Evolution and Characterization of the RNA Interference Pathways in Lophotrochozoa
Source: Genome Biol Evol. 2024 May 7;16(5):evae098. doi: 10.1093/gbe/evae098 (PMC11114477; doi:10.1093/gbe/evae098)
Supplement: evae098_Supplementary_Data [file evae098_supplementary_data.zip › Supplementary_materials.zip!/selected_miRNAs/NoGe_selected_miRNAs/mirdeep_pdf_files/NMRB01002591.1_Notospermus_geniculatus_strain_Ushimado_scaffold2591,_whole_genome_shotgun_sequence_39379.pdf]

Provisional ID	:	NMRB01002591.1_Notospermus_geniculatus_strain_Ushimado_scaffold2591,_whole_genome_shotgun_sequence_39379
Score total	:	3428.3
Score for star read(s)	:	3.9
Score for read counts	:	3421.1
Score for mfe	:	0.3
Score for randfold	:	0
Score for cons. seed	:	3
Total read count	:	6722
Mature read count	:	5938
Loop read count	:	0
Star read count	:	784

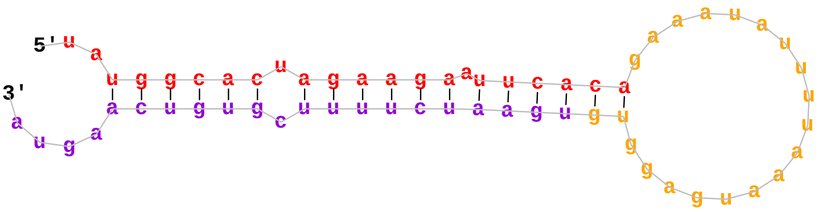

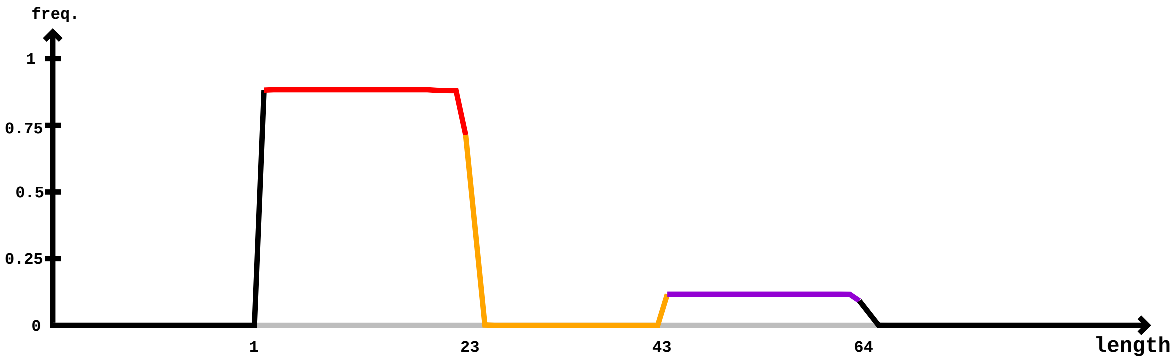


```
                         Mature                                       Star
5'- uuuagaaaguguugucugucuauggcacuagaagaauucacagaaauauuuuaaaugagguguggaaucuuuucgugucaaguacagauugcuccgcuugcuucacgaacuuc   -3'    obs
    uuuagaaaguguugucugucuauggcacuagaagaauucacagaaauauuuuaaaugaggugugaaucuuuucgugucaaguacagauugcuccgcuugcuucacgaacuuc          exp
    ...((.(((((..((((((((.((((((.((((((.((((((...................)))))))))))).)))))))).))))))).....))))).))..........   reads   mm   sample
    ....................uauggcacuagaagaauGcac.......................................................................   1       1    Fm1
    ....................uauggcacuagaagaaGucac.......................................................................   2       1    Fm1
    ....................uauggcacuagaagaauucac.......................................................................   78      0    Fm1
    ....................uauggcacuaAaagaauucaca......................................................................   2       1    Fm1
    ....................Nauggcacuagaagaauucaca......................................................................   1       1    Fm1
    ....................uauggcacuagaagaauCcaca......................................................................   6       1    Fm1
    ....................uauggcacuagaagaaGucaca......................................................................   2       1    Fm1
    ....................uauggcacuagaagaauucacG......................................................................   1       1    Fm1
    ....................uaugAcacuagaagaauucaca......................................................................   1       1    Fm1
    ....................uauggcacuagaagaauucaca......................................................................   469     0    Fm1
    ....................uauggcacuagaagaaAucaca......................................................................   1       1    Fm1
    ....................uauggcacuGgaagaauucaca......................................................................   1       1    Fm1
    ....................uauggcacuaUaagaauucaca......................................................................   1       1    Fm1
    ....................uauggcacuagaagaauAcaca......................................................................   3       1    Fm1
    ....................uauggcacuagaagaauGcaca......................................................................   2       1    Fm1
    ....................uauggcacuagaagaauucacaA.....................................................................   15      1    Fm1
    ....................uauggcacuagaagaauucacaU.....................................................................   2       1    Fm1
    ....................uauggcacuagaagaauucacaAa....................................................................   1       1    Fm1
    .....................auggcacuagaagaauucac.......................................................................   1       0    Fm1
    ..............................................................ugaaucuuuucgugucaagu..............................   8       0    Fm1
    ..............................................................ugaaucuuuuGgugucaagu..............................   1       1    Fm1
    ..............................................................ugaaucuuuucgugucCagu..............................   1       1    Fm1
    ..............................................................ugaaucuuuucgugucaagua.............................   49      0    Fm1
    ..............................................................ugaaCcuuuucgugucaagua.............................   1       1    Fm1
    ..............................................................ugaaucuuuucgugucGagua.............................   1       1    Fm1
    ..............................................................Agaaucuuuucgugucaagua.............................   1       1    Fm1
    ..............................................................ugaaucuuuucgugucaaguaU............................   23      1    Fm1

    ....................uauggcacuagaagaaAucac.......................................................................   1       1    Fm2
    ....................uauggcacuagaagaauCcac.......................................................................   1       1    Fm2
    ....................uauggcacuagaagaauAcac.......................................................................   4       1    Fm2
    ....................uauggcacuagaagaauucac.......................................................................   127     0    Fm2
    ....................uauggcacuagaaCaauucaca......................................................................   1       1    Fm2
```

```
                    Mature                                      Star
uuuagaaaguguugucugucuauggcacuagaagaauucacagaaauauuuuaaaugaggugugaaucuuuucgugucaaguacagauugcuccgcuugcuucacgaacuuc
....................Nauggcacuagaagaauucaca......................................................................   1      1   Fm2
....................uauggcacuagaagaauucaca......................................................................   821    0   Fm2
....................uauggcacuGgaagaauucaca......................................................................   1      1   Fm2
....................uauggcUcuagaagaauucaca......................................................................   1      1   Fm2
....................uauggcacuaAaagaauucaca......................................................................   1      1   Fm2
....................uauggcacuagUagaauucaca......................................................................   1      1   Fm2
....................uauggcacuagaGgaauucaca......................................................................   1      1   Fm2
....................uauggcacuagaagaaCucaca......................................................................   6      1   Fm2
....................uauggcacuagaCgaauucaca......................................................................   1      1   Fm2
....................uauggcacuagaagaaGucaca......................................................................   1      1   Fm2
....................uauggcacuagaagaauCcaca......................................................................   5      1   Fm2
....................uauggcacuagaagaauGcaca......................................................................   2      1   Fm2
....................uauggcacuagaagaauAcaca......................................................................   2      1   Fm2
....................uaAggcacuagaagaauucaca......................................................................   1      1   Fm2
....................uauggcacuagaagaauuUaca......................................................................   1      1   Fm2
....................uaGggcacuagaagaauucaca......................................................................   1      1   Fm2
....................uauggcCcuagaagaauucaca......................................................................   1      1   Fm2
....................uauggcacuGgaagaauucacag.....................................................................   1      1   Fm2
....................uauggcacuagaagaauucacaA.....................................................................   56     1   Fm2
....................uauggcacuagaagaauucacaC.....................................................................   1      1   Fm2
....................uauggcacuagaagaauucacaAa....................................................................   5      1   Fm2
..............................................................ugaaucuuCucgugucaagua.............................   1      1   Fm2
..............................................................ugaaucuuuucgugucaagua.............................   32     0   Fm2
..............................................................ugaaucuuuucgugucaaguaU............................   34     1   Fm2

....................uauggcacuagaagaauA..........................................................................   1      1   Mm1
....................uauggcacuagaagaauu..........................................................................   12     0   Mm1
....................uauggcacuagaagaauucac.......................................................................   51     0   Mm1
....................uauggcacuagaagaauAcac.......................................................................   1      1   Mm1
....................uauggcacuagaagaauucacU......................................................................   5      1   Mm1
....................uauggcacuaUaagaauucaca......................................................................   1      1   Mm1
....................uauggcacuaAaagaauucaca......................................................................   2      1   Mm1
....................uauggcacuagaagaauGcaca......................................................................   2      1   Mm1
....................uauggcacuagaagaauuAaca......................................................................   1      1   Mm1
....................uauggcacuagaGgaauucaca......................................................................   1      1   Mm1
....................uauggcacuagaagGauucaca......................................................................   1      1   Mm1
....................uauggcacuagaagaauAcaca......................................................................   1      1   Mm1
....................uauggcacuagaagaauucaca......................................................................   397    0   Mm1
....................uauggcCcuagaagaauucaca......................................................................   1      1   Mm1
....................uauggUacuagaagaauucaca......................................................................   5      1   Mm1
....................uauggcacuagaagaaGucaca......................................................................   2      1   Mm1
....................uauggcacuagaagaaCucaca......................................................................   1      1   Mm1
....................uauggcacuagaagaaAucaca......................................................................   1      1   Mm1
....................uauggcacuagaagaauucacaA.....................................................................   42     1   Mm1
.....................auggcacuagaagaauucaca......................................................................   1      0   Mm1
..............................................................ugaaucuuuucgugucaagu..............................   39     0   Mm1
..............................................................ugaaucuuuucgugucaagua.............................   44     0   Mm1
..............................................................ugaaucuuuucgugucaaguaU............................   10     1   Mm1

....................uauggcacuagaagaauuc.........................................................................   3      0   Mm2
....................uauggcacuagaagaauucUc.......................................................................   1      1   Mm2
....................uauggcacuaUaagaauucac.......................................................................   1      1   Mm2
....................uauggcacuagaagaaCucac.......................................................................   1      1   Mm2
....................uauggcacuagaagaauCcac.......................................................................   8      1   Mm2
....................uauggcacuagaagaauAcac.......................................................................   1      1   Mm2
....................uauggcacuaCaagaauucac.......................................................................   2      1   Mm2
....................uauggcacuagaaAaauucac.......................................................................   1      1   Mm2
....................uauggcacuagaagaauucac.......................................................................   468    0   Mm2
....................uauggcacuagaagaauGcac.......................................................................   3      1   Mm2
....................uauggcUcuagaagaauucac.......................................................................   1      1   Mm2
....................uaugAcacuagaagaauucaca......................................................................   1      1   Mm2
....................uauggcacuagaagaaGucaca......................................................................   4      1   Mm2
....................uauggcacuagaagaauuUaca......................................................................   2      1   Mm2
....................uauggcacuagaagaauGcaca......................................................................   10     1   Mm2
....................uauggcacuagaagaauucacU......................................................................   3      1   Mm2
....................uauggcacuagaagaaCucaca......................................................................   7      1   Mm2
....................uauggcUcuagaagaauucaca......................................................................   1      1   Mm2
....................uauggcacuagaagaauucaca......................................................................   1719   0   Mm2
....................Cauggcacuagaagaauucaca......................................................................   1      1   Mm2
....................uauggcacuagaagaauAcaca......................................................................   11     1   Mm2
```

```
                    Mature                                    Star
uuuagaaaguguugucugucuauggcacuagaagaauucacagaaauauuuuaaaugaggugugaaucuuuucgugucaaguacagauugcuccgcuugcuucacgaacuuc
....................uauggcacuagaagaauucacG......................................................................    2     1     Mm2
....................uauggcacuaAaagaauucaca......................................................................    5     1     Mm2
....................uauggcacuagaagaauucUca......................................................................    1     1     Mm2
....................uauggcacuagaagaaAucaca......................................................................    3     1     Mm2
....................uauggcacuaUaagaauucaca......................................................................    3     1     Mm2
....................uauggNacuagaagaauucaca......................................................................    1     1     Mm2
....................uauggcacuagaagaauCcaca......................................................................    14    1     Mm2
....................uauggcacuagaagaUuucaca......................................................................    1     1     Mm2
....................uauggcacuGgaagaauucaca......................................................................    2     1     Mm2
....................uauggcacuagaagaauucacaA.....................................................................    106   1     Mm2
....................uauggcacuagaagaauucacag.....................................................................    2     0     Mm2
....................uauggcacuagaagaauucacaU.....................................................................    10    1     Mm2
....................uauggcacuagaagaauucacaAa....................................................................    1     1     Mm2
.....................Uuggcacuagaagaauucac.......................................................................    1     1     Mm2
.....................auggcacuagaagaauucaca......................................................................    3     0     Mm2
..............................................................ugaaucuuuucgugucaag...............................    2     0     Mm2
..............................................................ugaaucuuuucgugucCagu..............................    1     1     Mm2
..............................................................ugaaucuuuucgugucaagu..............................    46    0     Mm2
..............................................................ugaaucuuuucgugucaagG..............................    2     1     Mm2
..............................................................ugaaCcuuuucgugucaagu..............................    1     1     Mm2
..............................................................ugaaAcuuuucgugucaagua.............................    1     1     Mm2
..............................................................ugaaucuuuucgugucUagua.............................    1     1     Mm2
..............................................................ugaaucuuuucgugucaaguU.............................    1     1     Mm2
..............................................................Agaaucuuuucgugucaagua.............................    1     1     Mm2
..............................................................ugaaucuuuucgugucaUgua.............................    1     1     Mm2
..............................................................ugaaucuuuucgugucaagua.............................    215   0     Mm2
..............................................................ugaaucuuuucgugucaaguaA............................    12    1     Mm2
..............................................................ugaaucuuuucgugucaaguaU............................    108   1     Mm2
..............................................................ugaaucuuuucgugucaaguaAa...........................    1     1     Mm2

....................uauggcacuagaagaauu..........................................................................    2     0     Fm3
....................uauggcacuagaagaauuc.........................................................................    1     0     Fm3
....................uauggcacuagaagaauuca........................................................................    1     0     Fm3
....................uauggcacuagaagaaAucac.......................................................................    2     1     Fm3
....................uauggcacuaCaagaauucac.......................................................................    1     1     Fm3
....................uauggcacuagaagaauCcac.......................................................................    1     1     Fm3
....................uCuggcacuagaagaauucac.......................................................................    1     1     Fm3
....................uauggcacuagaagaauAcac.......................................................................    1     1     Fm3
....................uauggcacuagCagaauucac.......................................................................    1     1     Fm3
....................uauggcacuaUaagaauucac.......................................................................    1     1     Fm3
....................uauggcacuagaUgaauucac.......................................................................    1     1     Fm3
....................uauggcacuaAaagaauucac.......................................................................    2     1     Fm3
....................uauggcacuagaagaauucac.......................................................................    252   0     Fm3
....................uauggcacuagaagaaGucac.......................................................................    1     1     Fm3
....................uauggcacuagaagaauGcac.......................................................................    3     1     Fm3
....................uauggcacuaUaagaauucaca......................................................................    1     1     Fm3
....................uauggcacGagaagaauucaca......................................................................    1     1     Fm3
....................uauAgcacuagaagaauucaca......................................................................    1     1     Fm3
....................uauggNacuagaagaauucaca......................................................................    1     1     Fm3
....................Nauggcacuagaagaauucaca......................................................................    1     1     Fm3
....................uauggcacuagaagaaGucaca......................................................................    3     1     Fm3
....................uauggcacuagaagaauAcaca......................................................................    6     1     Fm3
....................Aauggcacuagaagaauucaca......................................................................    1     1     Fm3
....................uauggcacuagaagaauuUaca......................................................................    1     1     Fm3
....................uauggcacuagUagaauucaca......................................................................    1     1     Fm3
....................uauggcacuagaagaaAucaca......................................................................    3     1     Fm3
....................Cauggcacuagaagaauucaca......................................................................    5     1     Fm3
....................uauggcacuagaagaauucacU......................................................................    4     1     Fm3
....................uauggcacuagaagaauGcaca......................................................................    5     1     Fm3
....................uauggcacuagaagaauCcaca......................................................................    5     1     Fm3
....................uauggcacuagaagaaCucaca......................................................................    3     1     Fm3
....................uauggcacuagaagaauucaca......................................................................    640   0     Fm3
....................uauggcacuagaagaauucacaA.....................................................................    10    1     Fm3
....................uauggcacuagaagaauucacaU.....................................................................    3     1     Fm3
.....................auggcacGagaagaauucac.......................................................................    1     1     Fm3
.....................auggcacuagaagaauucaca......................................................................    2     0     Fm3
.....................auggcacuagaagaauucacaga....................................................................    1     0     Fm3
..............................................................ugaaucuuuucgugucUagu..............................    1     1     Fm3
..............................................................ugaaucuuuucgugucaagu..............................    50    0     Fm3
..............................................................ugaaucuuuucgugucaagua.............................    47    0     Fm3
```

Mature Star

```
uuuagaaaguguugucugucuauggcacuagaagaauucacagaaauauuuuaaaugaggugugaaucuuuucgugucaaguacagauugcuccgcuugcuucacgaacuuc
..............................................................ugaaucuuuucguCucaagua.............................   1    1   Fm3
..............................................................ugaaucuuuucgugucaaguaU............................   12   1   Fm3

....................uauggcacuagaagaauu..........................................................................   1    0   Mm3
....................uauggcacuagaagaauuc.........................................................................   1    0   Mm3
....................uauggcacuagaagaauuca........................................................................   1    0   Mm3
....................uauggcacuagaagaauCcac.......................................................................   1    1   Mm3
....................uauggcacuagaagaauucac.......................................................................   94   0   Mm3
....................uauggcacuagaagaaGucac.......................................................................   1    1   Mm3
....................uauggcacuagaagaauucaca......................................................................   283  0   Mm3
....................uauggcacuagaagaaAucaca......................................................................   2    1   Mm3
....................uauggcacuagaagaauucGca......................................................................   1    1   Mm3
....................uauggcacuaCaagaauucaca......................................................................   1    1   Mm3
....................uauggcacuagaagaauuUaca......................................................................   1    1   Mm3
....................uauggcacuagaagaauucacU......................................................................   1    1   Mm3
....................uauggcacuagaagaauucacaU.....................................................................   1    1   Mm3
....................uauggcacuagaagaauucacaA.....................................................................   10   1   Mm3
....................uauggcacuagaagaauucacaAa....................................................................   1    1   Mm3
..............................................................ugaaucuuuucgugucCagu..............................   1    1   Mm3
..............................................................ugaaucuuuucgugucaagu..............................   12   0   Mm3
..............................................................ugaaucuuuucgugucUagu..............................   1    1   Mm3
..............................................................ugaaucuuuucgugucaagua.............................   12   0   Mm3
..............................................................ugaaucuuuucgugucaaguaA............................   4    1   Mm3
..............................................................ugaaucuuuucgugucaaguaU............................   5    1   Mm3
```
